# Patient-centered recruitment and retention for a randomized controlled study

**DOI:** 10.1186/s13063-018-2578-7

**Published:** 2018-03-27

**Authors:** Sumedha Chhatre, Ashlie Jefferson, Ratna Cook, Caitlin R. Meeker, Ji Hyun Kim, Kayla Marie Hartz, Yu-Ning Wong, Adele Caruso, Diane K. Newman, Knashawn H. Morales, Ravishankar Jayadevappa

**Affiliations:** 1 Department of Psychiatry, Perelman School of Medicine, University of Pennsylvania, 3535 Market St. Suite 4051, Philadelphia, PA 19104 USA; 2 Department of Medicine, Perelman School of Medicine, University of Pennsylvania, Philadelphia, PA USA; 3 Fox Chase Cancer Center, Temple University Health System, Philadelphia, PA USA; 4 Edward Via College of Osteopathic Medicine, Blacksburg, VA USA; 5 Division of Urology, Department of Surgery, Perelman School of Medicine, University of Pennsylvania, Philadelphia, PA USA; 6 Department of Biostatistics and Epidemiology, Perelman School of Medicine, University of Pennsylvania, Philadelphia, PA USA; 7 Corporal Michael J. Crescenz VAMC, Philadelphia, PA USA; 8 Leonard Davis Institute of Health Economics, University of Pennsylvania, Philadelphia, PA USA; 9 Abramson Cancer Center, University of Pennsylvania, Philadelphia, PA USA

## Abstract

**Background:**

Recruitment and retention strategies for patient-centered outcomes research are evolving and research on the subject is limited. In this work, we present a conceptual model of patient-centered recruitment and retention, and describe the recruitment and retention activities and related challenges in a patient-centered comparative effectiveness trial.

**Methods:**

This is a multicenter, longitudinal randomized controlled trial in localized prostate cancer patients.

**Results:**

We recruited 743 participants from three sites over 15 months period (January 2014 to March 2015), and followed them for 24 months. At site 1, of the 773 eligible participants, 551 (72%) were enrolled. At site 2, 34 participants were eligible and 23 (68%) enrolled. Of the 434 eligible participants at site 3, 169 (39%) enrolled. We observed that strategies related to the concepts of trust (e.g., physician involvement, ensuring protection of information), communication (e.g., brochures and pamphlets in physicians’ offices, continued contact during regular clinic visits and calling/emailing assessment), attitude (e.g., emphasizing the altruistic value of research, positive attitude of providers and research staff), and expectations (e.g., full disclosure of study requirements and time commitment, update letters) facilitated successful patient recruitment and retention. A stakeholders’ advisory board provided important input for the recruitment and retention activities. Active engagement, reminders at the offices, and personalized update letters helped retention during follow-up. Usefulness of telephone recruitment was site specific and, at one site, the time requirement for telephone recruitment was a challenge.

**Conclusions:**

We have presented multilevel strategies for successful recruitment and retention in a clinical trial using a patient-centered approach. Our strategies were flexible to accommodate site-level requirements. These strategies as well as the challenges can aid recruitment and retention efforts of future large-scale, patient-centered research studies.

**Trial registration:**

Clinicaltrials.gov, ID: NCT02032550. Registered on 22 November 2013.

## Background

The cornerstone of a clinical trial is the willingness of participants to invest time and effort. However, recruitment and retention for clinical trials is challenging [1–5]. In the context of a research study, recruitment is the process through which an individual is recruited as a study participant. This process involves presenting the study details to the potential participant; review of the details (by the potential participant); making a decision about participating; and providing informed consent if the participants agrees to enroll in the study. Participant retention is the continuous engagement of the participant in the research study, post enrollment [6]. While achieving the required sample size for recruitment and retention is critical for robust statistical power, and study validity [2, 7–10], achieving the targeted sample in terms of number or timespan is challenging [2, 5, 7, 10–19]. It was reported that only 55% of clinical studies accrue their total targeted sample size, and 78% accrue 80% of their targeted sample size [18]. Recruitment and retention issues can adversely affect the ability to detect intervention effects and may limit the significance of the research findings [2, 5, 7, 20].

Patient-centered outcomes research (PCOR) is a way to achieve patient-centeredness in research. Patient-centeredness requires active participation and can lead to empowerment [21, 22]. Focus of PCOR is on comparing the effects of treatment options on outcomes that matter most to the patients [21, 23]. Increased participation of stakeholders in clinical trials is a key element of PCOR that can enhance concordance between research topics and patient priorities, improve patient recruitment and retention, and inform clinically relevant evidence-based policy [21–24].

Community-based participatory research (CBPR) is an excellent model for engaging communities in research [25, 26]. There is an opportunity for the PCOR model to be informed by CBPR principles for enhancing patient engagement, and the two models can complement each other [25, 27]. Principles of CBPR can be used to inform the three phases of a PCOR study: pre, continuous and sustained phase. The CBPR principles of community as a unit of identity, building on community strengths and equal partnerships can inform the pre-engagement phase of the PCOR research. The CBPR principles of bilateral learning and integration of research and action can inform the continuous engagement phase of PCOR research; and finally, the CBPR principles of system development, and involvement of all partners in the dissemination of findings can inform the sustained engagement phase of PCOR research.

Research indicates that recruitment strategies that evolve and adapt to the needs of the study and use local resources appropriately are successful [28]. In addition, successful strategies are those that enhance a participant’s awareness of the health issue [2, 29–32]. At the same time, operationalization of these insights into research protocols is challenging. Furthermore, while important, these studies do not describe a patient-centered approach for recruitment and retention. Patient-centered recruitment and retention implies continuous engagement of stakeholders to identify the resources available, develop appropriate strategies and address the challenges to recruitment and retention. The objective of our paper is twofold. First, we present a conceptual model of patient-centered recruitment and retention. Next, in the context of this conceptual model, we describe the recruitment and retention strategies, and related challenges in our PCOR-based comparative effectiveness randomized controlled trial (RCT) in localized prostate cancer patients.

## Methods

### Conceptual model of patient-centered recruitment and retention

**Fig. 1:**
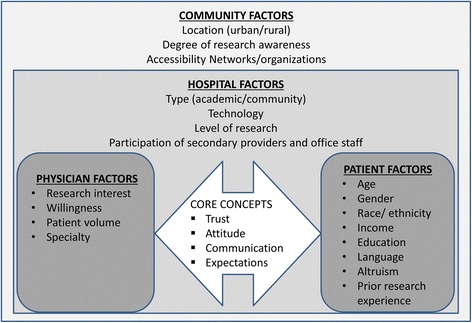
Conceptual model of patient-centered recruitment and retention

Our conceptual model of patient-centered recruitment and retention (Fig. 1) was developed by the study principal investigator prior to study initiation, and is informed by the PCOR principals [22, 25, 27, 33]. The model identifies four levels of factors (patient, physician, hospital, and community) that contribute to the engagement, recruitment and retention of participants in clinical trials. Community and hospital are the macro-level factors that influence feasibility, accessibility and translation of research. Patient and provider are micro-level factors that affect the conduct of, and engagement in, research. Trust, attitude, communication, and expectations between patients and providers are the core concepts of our model. A discussion of each core concept follows.

#### Trust

Trust is the foundation of recruitment and retention in clinical trials [32, 34–37]. Trust in physician was cited as a reason for participating in a study [32, 35]. When physicians advise patients about clinical research, the physician-patient relationship is enhanced [38, 39]. Patients may seek their physicians’ opinion about entry into a study [38], or the physician may refer the patient to a study. Either way, the patient may not fully assess the pros and cons of participation out of trust in the physician. Therefore, the physician needs to inform the patient about the optional nature of research participation and that the patients’ decision has no implications for care [38, 40].

#### Attitude

Overall attitude of the patient and provider has implications for recruitment and retention in a study [5]. Altruistic attitude of participants was a main motivator for study participation [35, 37]. Physician’s attitude towards research was cited as a barrier to participation by some [32, 41].

#### Communication

An important principle of PCOR, communication or engagement, plays a vital role in recruitment and retention [5, 23, 33]. Patients must receive information that is useful for making health decisions. In a study of 485 consecutive patients in a tertiary-care academic medical center, over two thirds of participants said that it was appropriate for an investigator to contact them about a study [5]. Patients also reported that they liked to receive details of the study [42]. Research protocols must describe the specifics about health questions that the research aims to inform; and the relevant population [23]. Clear communication about study requirements is shown to improve study retention [43].

#### Expectations

Participants have certain expectations regarding the research they participate in. Participants expected to be briefed about study outcomes upon its completion, and said it would affect their decision about future participation [5, 42]. Lack of incentives was noted as a barrier to recruitment [44], whereas being responsive to the needs of the participants and developing a rapport led to successful recruitment [44].

#### Process of developing recruitment and retention strategies

Our study advisory board comprised of multiple stakeholders – prostate cancer survivors, providers and researchers. During regular meetings with the study team, based on their personal and clinical experience, the stakeholders were able to offer important insight into the essential steps (identification of potential participants, contacting the potential participants, explaining the study, obtaining informed consent, conducting assessments and ongoing retention) and resources (personnel) that were necessary for participant recruitment and retention. Integration of these elements led to our recruitment and retention strategies that were rooted in the four core concepts of trust, attitude, communication and expectations.

### Study description and intervention

The overall study methodology for our study funded by the Patient Centered Outcomes Research Institute (PCORI) has been described previously [45]. In this multicentered RCT, the intervention was a web-based Adaptive Conjoint Analysis (ACA) tool, PreProCare for preferences assessment. Participants in the intervention group completed the web-based ACA tool, PreProCare tool, to assess their individual preferences. Briefly, in this three-part tool, brief introduction to the tool was provided in part 1. In the second part, the participants ranked the attributes of various treatments (“not important” to “extremely important”). In the third part, choice scenarios consisting of combinations of attributes were presented based on attributes ranking, with participants selecting the combination that they most prefer. At end of the task, a graph and a list of the five attributes most preferred by the participant was generated. The participant had the option to have a printout of the output to share with his provider [45]. The intervention group completed the intervention either during their office visit or at home. On average, this tool required about 30 min to complete. Usual care group participants received care as usual that consisted of standard educational material about prostate cancer treatments.

#### Sample size

Sample size analysis indicated that we needed a total of 720 participants, eligible for randomization into one of the two study groups to have 80% power for detecting an effect size as small as 0.2 standard deviations (SD) in outcomes. All participants were followed-up for 2 years and outcomes were assessed at baseline, at and 3-, 6-, 12-, and 24-month follow-up.

#### Randomization

The study biostatistician (KM) created randomization sequences for each site using a pseudo-random number generator with random blocking varying sizes from 2 to 6. The treatment assignments were placed in sealed, opaque envelopes. Research coordinators opened the envelope and notified participants of group assignment. Study investigators were masked to the treatment assignment.

#### Outcomes

Outcomes were satisfaction with care, satisfaction with decision, health-related quality of life, depression and anxiety, and treatment choice. Self-reported data on patient age, income, race, ethnicity, education, marital status, and employment status were obtained at baseline. Patient-reported data on control preference, decision conflict and trust were also assessed at baseline. Participants were offered a US$20 gift card at each assessment, as a token of appreciation. Local institutional review boards approved the study.

### Study sites

The University of Pennsylvania (site 1) was the primary and coordinating site for this protocol and four outpatient offices facilitated the accrual efforts. Other study sites were the Corporal Michael J. Crescenz Veterans Administration Medical Center (CMCVAMC) site 2) and Fox Chase Cancer Center (FCCC) and Temple Healthcare System (site 3).

### Study eligibility criteria

The study eligibility criteria were as follows: (1) newly diagnosed with localized prostate cancer (and yet to initiate curative intent treatment for prostate cancer) or under the active surveillance protocol; (2) age ≥ 18 years; (3) low risk (prostate-specific antigen (PSA) ≤ 10 ng/ml, and Gleason ≤ 6, and stage T1c–T2a), intermediate risk (PSA > 10 to ≤ 20 ng/ml, or Gleason 7, or stage T2b), and high risk (PSA > 20 ng/ml, or Gleason score 8–10, or stage T2c); (4) able to provide informed consent. The exclusion criteria are: (1) distant, metastatic or un-staged prostate cancer at diagnosis; (2) unable to communicate in English; and (3) already treated for prostate cancer with curative intent.

### Analysis

We summarized the patient-centered recruitment and retention strategies in the context of the four concepts. We also compared the baseline demographic and clinical characteristics of the intervention group and usual care groups.

## Results

### Core concepts, recruitment and retention strategies

Table 1 summarizes our patient-centered recruitment and retention strategies and the related core concepts. Initial and continued involvement of physicians in the study was essential for developing trust. Introduction by the physician helped the research staff to develop better rapport with the participant. Providing details about protection of health information to the participants was another element that developed trust. Positive attitude of providers and research staff was essential for our recruitment and retention efforts. During the engagement and recruitment process, our research team was mindful about describing the study protocol in a way that patients would understand. We also emphasized the altruistic value of research involvement. Our core concept of communication implied ongoing engagement of participants and was facilitated by continued contact by research coordinators during regular clinic visits, and calling and re-sending assessment packages to those unresponsive. The core value of expectations translated into full disclosure of study requirements and time commitment. Two sites sent update letters to the participants that briefed them about study progress, and thanked them for their continued participation. Advisory board members were also kept abreast of the study progress during regular advisory board meetings. Finally, at each study time point, participants were offered a gift card as a way of showing our appreciation. In the following sections, we present the details of the participant recruitment.

**Table 1:** Patient-centered values and strategies

Patient-centered core concepts	Recruitment strategies	Retention strategies
Trust	• Physician involvement and knowledge of the study ° Physician introduces patient to study and to research staff • Ensure patient protection of health information and identity ° Utilization of unique ID numbers instead of identifiers	• Upkeep physicians ongoing involvement ° Physician remains up to date with study to answer any questions • Assure easy accessibility to research staff
Attitude	• Encourage physicians to educate patients about importance of this research • Emphasize altruistic value of study • Foster encouragement from family members • Positive attitudes of clinicians, office staff, and research staff towards study • Previous experience with research studies • Systematic preparation by research staff prior to recruitment	Personalized handwritten thank-you notes for continued participation to maintain high interest over the entire study duration
Communication	• Use of brochures and flyers in hospitals and clinics of physicians ° Easily understandable • Effective communication when recruiting in person and via telephone ° Personality, professionalism, full knowledge of study • Easily accessible research materials and staff ° Websites with study aims and contact list	• Remind nonresponsive participants ° Contact via preferred method of communication (telephone or email) ° Make telephone calls during optimal hours of between 4 pm and 6 pm ° Re-sending assessment packages
Expectations	• Provide detailed but concise explanation of study purpose ° Full disclosure of study requirements and time commitment • Convey physicians’ interest in the research • Introduce compensatory incentives ° Provide gift cards at baseline and at follow-up completion to appreciate participation	• Engage stakeholders’ advisory board via regular meetings • Provide updates on study ° Send letters of progress reports • Hold weekly meetings with research staff across all sites ° Discuss updated retention numbers and progress ° Resolve any challenges faced

#### Onsite recruitment

Overall, the recruitment process consisted of five main steps: research coordinators reviewed medical records of patients scheduled for prostate needle biopsies, outpatient office appointments (urology, radiation oncology) for newly diagnosed prostate cancer or regularly scheduled active surveillance visits. Potentially eligible patients were given a unique identification number and tracked using a secure database. Prior to approaching the patients, we obtained approval from their providers. Physicians and nurse navigators also referred potential patients to the study. Each site adjusted these steps per site-specific needs.

#### Telephone recruitment

For telephone recruitment at site 1, we obtained a list of prostate cancer patients from Penn Medicine’s Clinical Data Warehouse, the Penn Data Store. Those diagnosed in the prior 6 months were eligible for telephone contact. Prior to recruitment, we obtained approval from urologists and radiation oncologists to contact their patients. During this time, we also amended the study protocol to include active surveillance as inclusion criteria. During telephone contact, research coordinators used an IRB-approved telephone script to screen the patients for any exclusion criteria, explain the study, and review the consent form. To those who verbally agreed to participate, we mailed two copies of the informed consent form along with a self-addressed, stamped envelope. Patients were instructed to read over the form carefully and if they wished to participate, sign and date the form and mail it back, and keep the second one for their records.

During study initiation at site 3, the site investigator discussed the study with the radiation oncologists and urologists at site 3, and received “blanket consent” to contact potential patients prior to their appointments. Potential participants were contacted via telephone prior to their upcoming appointment. Research staff used an IRB-approved telephone script to introduce the study. If agreeable, potential participants arrived 1 h early for their scheduled appointment to complete study-related activities. If a patient verbally agreed to participate, he was mailed two copies of the informed consent form along with pre-paid return envelope. In case of those who declined to participate, the reason for refusal was noted, and they were removed from the contact list. This strategy allowed staff to gauge the patient’s attitude toward participation, and to address the expectations of the participant, and what they could expect from the study team if they participated. If a potential participant was unreachable via telephone prior to his upcoming appointment, we attempted to approach him in the clinic. Physicians and nurse navigators were also encouraged to refer eligible patients to the study.

#### Retention and follow-up

To enhance the participants’ engagement and communication, at sites 1 and 2, we mailed a one-page study update to the participants during the follow-up period. Study coordinators also created lists of unresponsive participants, and the assessments they had missed. We shared these lists with the providers so that they could stress the importance of continued participation during a participant’s follow-up visit. At all sites, we re-sent the questionnaires to those who were nonresponsive. Additionally, telephone calls were made and emails were sent to those who could not be contacted during their office visits. Direct interactions, such as speaking to participants during a provider visit or over telephone, was helpful. We also observed that calling later in the afternoon, and/or early evening, after work hours, were the most appropriate times than calling in the morning or early afternoon. Given the large number of participants, effective tracking to monitor retention was crucial. Study coordinators developed a management system for all study activities, including a physical filing system with copies of letters that were sent and received, consent forms, copies of completed questionnaires, and any other correspondence, as well as a secured electronic database that included logging of individual randomization and retention-related information.

### Patient recruitment and retention

Using the strategies described in Table 1, between January 2014 and March 2015, we recruited 743 patients with localized prostate cancer from the three sites, and followed them for 24 months. Across three sites, we assessed 4558 patients for eligibility, and excluded 3317 for various reasons. At site 1, of the 773 eligible participants, 551 (72%) were enrolled. At site 2, 34 participants were eligible and 23 (68%) enrolled in the study. Of the 434 eligible men at site 3, 169 (39%) enrolled. In total, 743 patients were randomized (371 to intervention and 372 to usual care). The in-person recruitment among eligible patients was more effective than telephone recruitment (87% vs. 41%). The top three reasons for declining to participate were a feeling of being overwhelmed with the diagnosis, not being interested in a research trial, and just wanting to move on with life.

**Fig. 2:**
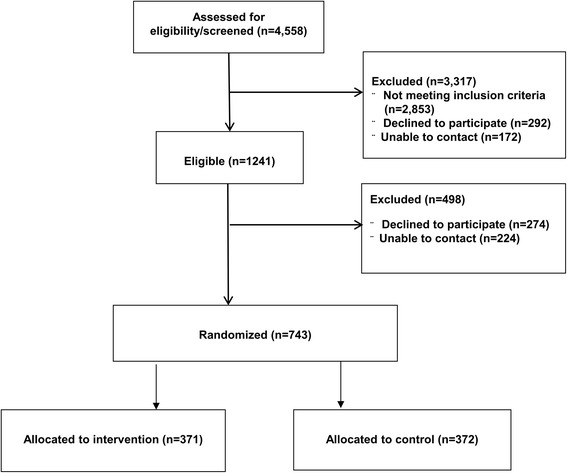
Consolidated Standards of Reporting Trials (CONSORT) Diagram

Table 2 presents the baseline characteristics of our study cohort. The mean age at diagnosis of prostate cancer was 63.58 years (SD 7.8). Most of the participants were white (79%), married (82%), with at least college degree (62%), and annual household income in excess of US$75,001 (61%). The intervention group and usual care group were comparable in terms of demographic and clinical characteristics. Figure 2 presents the Consolidated Standards of Reporting Trials (CONSORT) Diagram of patient recruitment. At 24 months, we maintained retention between 74 and 83% across all sites. It was observed that retention was higher was participants who were white, older than 75 years, and were married. To enhance the participants’ engagement, half way through the study course, we mailed a one-page study update to all participants at sites 1 and 2. A 35 % increase in survey response rate was observed following the dissemination of the letter, especially from participants who were otherwise unresponsive in responding to the surveys.

**Table 2:** Baseline demographic and clinical characteristics (*n* = 743)

Variables	All (*n* = 743)	Controls (*n* = 372)	Intervention (*n* = 371)	*P* value
Mean age at diagnosis (years) ± SD	63.6 ± 7.8	63.3 ± 7.6	63.8 ± 8.0	0.6345
Sites (*n*)				
Site 1	551	276	275	0.9855
Site 2	23	12	11	
Site 3	169	86	83	
Race/ethnicity (%)				
White	79.81	79.68	79.95	0.4055
African American	15.21	14.97	15.45	
Latino/Hispanic	0.94	1.34	0.54	
Asian	1.08	1.6	0.54	
Other	2.96	2.41	3.52	
Income (%), *n* = 551				
≤ 20,000	4.7	5.0	4.4	0.0561
20,001 to 40,000	10.3	11.9	8.8	
40,001 to 75,000	19.2	20.5	17.9	
≥ 75,000	60.6	59.4	61.9	
Unknown	5.1	3.2	6.9	
Marital status (%), *n* = 551				
Married	82.2	83.5	80.9	0.2064
Single/divorced/widowed	16.3	16.2	16.5	
Unknown	1.5	0.36	2.6	
Education (%), *n* = 551				
High school or above	18.0	18.3	16.8	0.38063
Some college	20.3	20.9	19.8	
College/advanced degree	61.7	60.8	62.6	
Missing	0.36	0	0.73	
Employment (%), *n* = 551				
Full-time	46.3	46.0	46.5	0.70692
Part-time	8.9	9.4	8.4	
Retired	38.5	40.3	36.6	
Unknown	6.4	4.3	8.4	
Average number of people in household	2.34	2.34	2.34	0.7666

## Discussion

Involvement of patient and other stakeholders’ in research is an essential component of PCOR. Additionally, the PCORI encourages stakeholders’ involvement over the entire research spectrum. Thus, for a patient-centered outcomes study, it is necessary that researchers have a collaborative relationship with both participants and stakeholders. Our work offers new insights into patient-centered recruitment and retention strategies in the context of a large, patient-centered RCT study in the following ways: (1) a novel patient-centered recruitment and retention model to operationalize concepts of trust, attitude, communication and expectations; (2) active engagement of all stakeholders including patients to enhance recruitment and retention; (3) sensitivity to the needs and challenges; and (4) appropriate use of available resources at study sites.

Our conceptual model of patient-centered recruitment and retention consists of patient-, physician-, hospital-, and community-level factors that have implications for recruitment and retention. We observed that strategies related to our concepts of trust (e.g., physician involvement, ensuring protection of information), communication (e.g., brochures and pamphlets in physicians’ offices, continued contact during regular clinic visits, resending assessments), attitude (e.g., emphasizing the altruistic value of research, positive attitude of providers and research staff), and expectations (e.g., full disclosure of study requirements and time commitment, update letters) facilitated recruitment and retention. Screening patient records, identifying eligible patients, preparing recruitment material and ensuring that the relevant clinicians were informed about the study, were useful strategies in our study, and have been identified by prior research [9, 28]. The in-person recruitment among eligible patients was more effective than telephone recruitment (87% vs. 41%). However, despite having trained research staff, time and resource constraints prompted use of diverse techniques to augment the recruitment and retention efforts. Contacting potential participants prior to their scheduled appointment helped streamline the process at one site. Participants arrived 1 h early for their appointment, and this offered sufficient time to discuss questions and concerns.

Collaboration between patients, clinicians, and researchers helped build trust. Most of our patients were in a sensitive period, having recently learned about their cancer diagnosis. The treating physician was fully knowledgeable about the study and the patients’ circumstances. The physician introduced the study and the research staff to the participant. Because of this process, the participant was able to appreciate the physicians’ involvement with the study. Additionally, the research staff discussed the measures that were in place for protection of health information and identity. The physician maintained ongoing involvement with the study and along with the research staff, remained accessible to the participant. Together, these measures generated trust and helped the recruitment and retention efforts. Studies report that up to 76% of patients expected their physician to alert them about appropriate clinical trials [31], and that physician referral was one of the most useful recruitment strategy [30]. One RCT of localized prostate cancer patients analyzed the comparative effectiveness and cost-effectiveness of nurses and surgeons in recruiting patients. The recruitment rates between urology consultants and nurses were not significantly different, and although nurses spent longer time with patients on average, they were more cost-effective recruiters [46]. Due to the uncertainty that is inherent in clinical research, time pressures and lack of information about study details, physicians may be reluctant to discuss clinical research with patients [38]. However, interaction between physician and patient is an important aspect of study recruitment [28], whereas medical distrust is often cited as a barrier to recruitment [11, 18, 20, 28].

Attitude was another of our core concept that aided recruitment and retention. By learning the flow of each physician’s office and working closely with the staff, we were able to enhance in-person recruitment. Altruism is one of the reasons for participating in a clinical trial [38]. Across all study sites, we observed that patients agreed to enroll after they learnt that study has the potential to help future prostate cancer patients. Many of the enrolled patients were approached fairly soon after learning about diagnosis and were usually accompanied by family members. While the family members were naturally protective of the patient initially, when presented with the opportunity to help future patients, the family members played a pivotal role in encouraging the patient to participate.

We encountered some challenges in the course of recruitment and retention activities. For example, identifying and recruiting eligible active surveillance patients was time consuming as it mostly involved telephone recruitment. Usefulness of telephone recruitment was site specific. While at site 3 it was helpful in reaching and recruiting patients, at site 1, it was challenging to contact the patients from Penn Data Store list. Often the patient requested a call back at a specific time, which sometimes was inconvenient. One study attempted recruitment using targeted telephone and mail lists, and found that calling was time consuming and inefficient, similar to our findings [12].

Research indicates that geographical location has an effect on recruitment for clinical trials, and that large cities were associated with poorer recruitment [9, 28, 47]. The likely reasons are a more diverse population, traditionally more difficult to engage subgroups, greater population mobility, and presence of multiple research entities resulting in participant exhaustion [9, 47]. Although recruitment for our study took place at urban, academic hospitals, we did not face these obstacles. While we attempted to recruit participants from all socio-economic groups, most of our participants had at least a college degree, and an annual household income of more than US$75,000.

Maintaining engagement at desired levels needs monitoring and tailored solutions for study-specific challenges [28]. In general population, the response rates for self-completion surveys was as little as 11% [47, 48]. It is likely that respondents have better health statuses, compared to nonrespondents and this can affect the generalizability of the results [47, 49, 50]. Our retention strategies were also rooted in our core concept of “expectations”, e.g., providing study updates, and offering monetary incentive. Research has identified participant incentive as a helpful strategy to enhance retention [51]. In a study of lung cancer patients, acknowledging and appreciating the efforts of participants motivated continued participation [51].

### Limitations

We note some limitations to our study. All three sites in our study are urban academic institutions and our sample consisted of men with localized prostate cancer. Most of our participants were college graduates, and had an annual household income of more than US$75,000. Thus, the generalizability of the findings may be limited. Additionally, of the four levels of factors (patient, provider, hospital, and community) depicted in our conceptual model, we focused mostly on the patient- and provider-level factors. There may be core concepts in addition to the ones that we have identified, and these need to be addressed in future studies. While we observed some differences in the yield of different strategies, it is not fully possible to separate the effects of different recruitment and retention approaches because several strategies were in place concurrently and no single strategy was adopted in isolation. Each research study faces specific conditions, and some recruitment and retention strategies we have presented may be more relevant than other strategies. Finally, in this paper our focus describing our novel patient-centered recruitment and retention activities. While we present the demographic differences between intervention and usual care groups to demonstrate that randomization worked, the outcomes data will be presented in our future work.

## Conclusion

We have presented recruitment and retention strategies within the context of a patient-centered model. Despite approaching patients, many of whom only recently had learned about diagnosis of prostate cancer and thus were definitely in a sensitive period, and the short window within which to recruit and administer the intervention, we were able to recruit 743 men by utilizing these multiple, proactive recruitment techniques. Additionally, our strategies were flexible to accommodate site-level requirements. Our strategies as well as challenges can aid recruitment and retention efforts of future large-scale, patient-centered research studies.

## Abbreviations

CBPR: Community-based participatory research
PCOR: Patient-centered outcomes research
PCORI: Patient Centered Outcomes Research Institute
RCT: Randomized controlled trial
SD: Standard deviation
